# A Summary of Organizations Providing Evidence-Based Dementia Caregiving Programs in Best Programs for Caregiving

**DOI:** 10.1093/geroni/igaf122.2214

**Published:** 2025-12-31

**Authors:** Zoe Fete, Megan Huth, David Bass, Rachel Cannon, Sara Powers, Morgan Minyo

**Affiliations:** Benjamin Rose, Cleveland, Ohio, United States; Benjamin Rose Institute on Aging, Cleveland, Ohio, United States; Benjamin Rose Institute on Aging, Cleveland, Ohio, United States; Benjamin Rose Institute on Aging, Cleveland, Ohio, United States; Benjamin Rose, Cleveland, Ohio, United States; Benjamin Rose Institute on Aging, Cleveland, Ohio, United States

## Abstract

Best Programs for Caregiving (BPC) is a free online resource of 45 evidence-based dementia caregiving programs. The Public Version of BPC, launched in 2024, enables family/friend caregivers to use a zip code search to find BPC programs available in their communities. Data were analyzed from a structured survey of 206 organizations identified by program developers as delivering their BPC program and used to populate the Public Version. The most common types of delivery organizations were healthcare or single-service community organizations (37; 18.0%), AAAs (37; 18.0%), multi-service community organizations (31; 15.0%), and senior centers/meal programs (28; 13.6%). There were 28 (13.6%) organizations offering a BPC program nationwide to caregivers living anywhere in the US, and 178 offering a program locally or statewide (86.4%). Most organizations were delivering a BPC program partially or fully remote, with only 34 (16.5%) exclusively in-person. An overwhelming majority were delivered exclusively by professionals (160; 77.7%) and were free (175; 85%). Nearly a quarter of programs (49; 23.8%) were adapted for caregivers identifying with diverse communities, most commonly Hispanic and Latino/Latina, Black/African American and LGBTQ. There were 73 (35.8%) programs delivered in languages other than English, with the most frequent offering being Spanish (69). Findings demonstrate that caregivers throughout the US, including those who identify with underserved populations or live in underserved areas, have evidence-based support programs available in their communities, with many being free. Findings also illustrate variation in the characteristics of programs, enabling caregivers to choose options that most closely match their preferences.

